# Virtual Reality Technology in Cognitive Rehabilitation Application: Bibliometric Analysis

**DOI:** 10.2196/38315

**Published:** 2022-10-19

**Authors:** Danni He, Shihua Cao, Yuchao Le, Mengxin Wang, Yanfei Chen, Beiying Qian

**Affiliations:** 1 School of Nursing Hangzhou Normal University Hangzhou China; 2 Nursing Department Hangzhou Normal University Qianjiang College Hangzhou China

**Keywords:** virtual reality, cognitive rehabilitation, bibliometric analysis, CiteSpace, gCLUTO, rehabilitation, cognitive disorder, visual content analysis

## Abstract

**Background:**

In recent years, with the development of computer science and medical science, virtual reality (VR) technology has become a promising tool for improving cognitive function. Research on VR-based cognitive training has garnered increasing attention.

**Objective:**

This study aimed to investigate the application status, research hot spots, and emerging trends of VR in cognitive rehabilitation over the past 20 years.

**Methods:**

Articles on VR-based cognitive rehabilitation from 2001 to 2021 were retrieved from the Web of Science Core Collection. CiteSpace software was used for the visual analysis of authors and countries or regions, and Scimago Graphica software was used for the geographic visualization of published countries or regions. Keywords were clustered using the gCLUTO software.

**Results:**

A total of 1259 papers were included. In recent years, research on the application of VR in cognitive rehabilitation has been widely conducted, and the annual publication of relevant literature has shown a positive trend. The main research areas include neuroscience and neurology, psychology, computer science, and rehabilitation. The United States ranked first with 328 papers, and Italy ranked second with 140 papers. Giuseppe Riva, an Italian academic, was the most prolific author with 29 publications. The most frequently cited reference was “Using Reality to Characterize Episodic Memory Profiles in Amnestic Mild Cognitive Impairment and Alzheimer’s Disease: Influence of Active and Passive Encoding.” The most common keywords used by researchers include “virtual reality,” “cognition,” “rehabilitation,” “performance,” and “older adult.” The largest source of research funding is from the public sector in the United States.

**Conclusions:**

The bibliometric analysis provided an overview of the application of VR in cognitive rehabilitation. VR-based cognitive rehabilitation can be integrated into multiple disciplines. We conclude that, in the context of the COVID-19 pandemic, the development of VR-based telerehabilitation is crucial, and there are still many problems that need to be addressed, such as the lack of consensus on treatment methods and the existence of safety hazards.

## Introduction

### Background

Cognition refers to the mental processes associated with acquiring knowledge and converting it into mental activities [1]. Cognitive function is composed of multiple cognitive domains such as memory, language comprehension ability, spatial orientation, executive ability, and computing power [2]. Cognitive dysfunction refers to a decline in one or more domains of cognition caused by various factors. The main symptoms include poor responsiveness, apathy, reduced proactive behavior, and short-term memory impairment, which in turn affects the individual’s activities of daily living [3].

Cognitive impairment increases stroke risk in older adults [4]. In the presence of a stroke, dementia onset might occur 10 years earlier, with up to one-third of persons who have experienced a stroke being diagnosed with dementia [5,6]. Therefore, it is crucial to explore treatments that could prevent or delay cognitive impairment. Owing to the limited effectiveness of pharmacological treatments, nonpharmacological interventions to treat cognitive deficits have been widely studied in recent years [7]. Several advantages of computer-assisted cognitive rehabilitation have been demonstrated, such as individualized, flexible, and economical programs that provide immediate feedback [8]. In this type of treatment, technologies such as robots, noninvasive brain stimulation, wearable systems, and neuroprosthetics were used. Among these, virtual reality (VR) shows great potential for neurorehabilitation [9].

A VR system can be defined as a highly interactive 3D digital media environment. Users can receive multisensory feedback, such as auditory, tactile, and visual feedback, in the simulated environment [10]. Developed in the 1990s and used as a tool to assess and treat diseases [11], VR has the advantage of providing an environment that simulates the sensation of the real world, combined with the situations and physical needs in daily lives [9]. Several studies have confirmed the effectiveness of VR in treating diseases such as anxiety, cognitive decline in older adults, and bulimia nervosa [12]. Jang et al [13] found that sensory feedback during VR training affected neuroplasticity and promoted brain reorganization. Optale et al [14] conducted a randomized controlled trial to examine the effectiveness of a VR intervention in older adults with memory deficits. The control group was treated with music therapy and the treatment group underwent 3 months of VR memory intervention. After completing the training series, the VR group showed significant improvements in memory tests, especially in long-term recall, with an effect size of 0.7 [14]. The study by Burdea et al [15] showed that individuals with chronic poststroke symptoms in the VR group achieved significant improvements in depression (effect size=0.75) and cognition (effect size=0.46; *P*<.05). These examples suggest that VR can be used to improve impaired cognitive functions.

### Research Problem and Aim

Bibliometrics is the cross-disciplinary science of quantitative analysis of all knowledge carriers using mathematical and statistical methods. It is a commonly used method to identify the development of a certain field [16]. The advantage of bibliometrics is that it can help scholars quickly grasp the research hot spots and development trends of a specific research area by analyzing citations, cocitations, distribution of countries or regions, authors, and journals in the research field [17]. The study by Keshner et al [18] applied topic modeling methods to 3141 publications included in the Web of Science (WoS), highlighting the research emphasis on VR in rehabilitation. Studies on VR-based cognitive rehabilitation have been published worldwide. However, there is little research that analyzes the scientific output in this field from a bibliometric and visualization perspective. Understanding the research status and hot spots in this field is of great significance for promoting the rehabilitation of disorders related to cognitive impairment. Therefore, based on the WoS Core Collection, this study comprehensively analyzed the research field to provide a reference for future research.

## Methods

### Data Sources and Search Strategy

In this study, only articles and reviews published in English between 2001 and 2021 were included. The data used in this study were downloaded from the WoS Core Collection. The search strategy was as follows: (1) topic (“reality” OR “VR”) AND (“cognition” OR “cognitive function” OR “cognitive impairment” OR “cognitive dysfunction” OR “cognitive rehabilitation”); (2) the document type was selected as “Article” OR “Review”; (3) the language was selected as “English”; (4) the dates of the search were from January 1, 2001, to December 20, 2021. In total, 1286 documents met the selection criteria.

### Screening Strategy

The structured approach by Kable et al [19] to searching and critiquing the research literature was used to guide and support this review. In this study, 2 reviewers (DH and YC) screened the search results independently, and the results were then cross-checked. Upon encountering any disagreements, a third researcher (SC) was consulted. All the researchers received training in document retrieval and screening by studying the textbook *Medical Literature Information Retrieval* [20].

**Figure 1:**
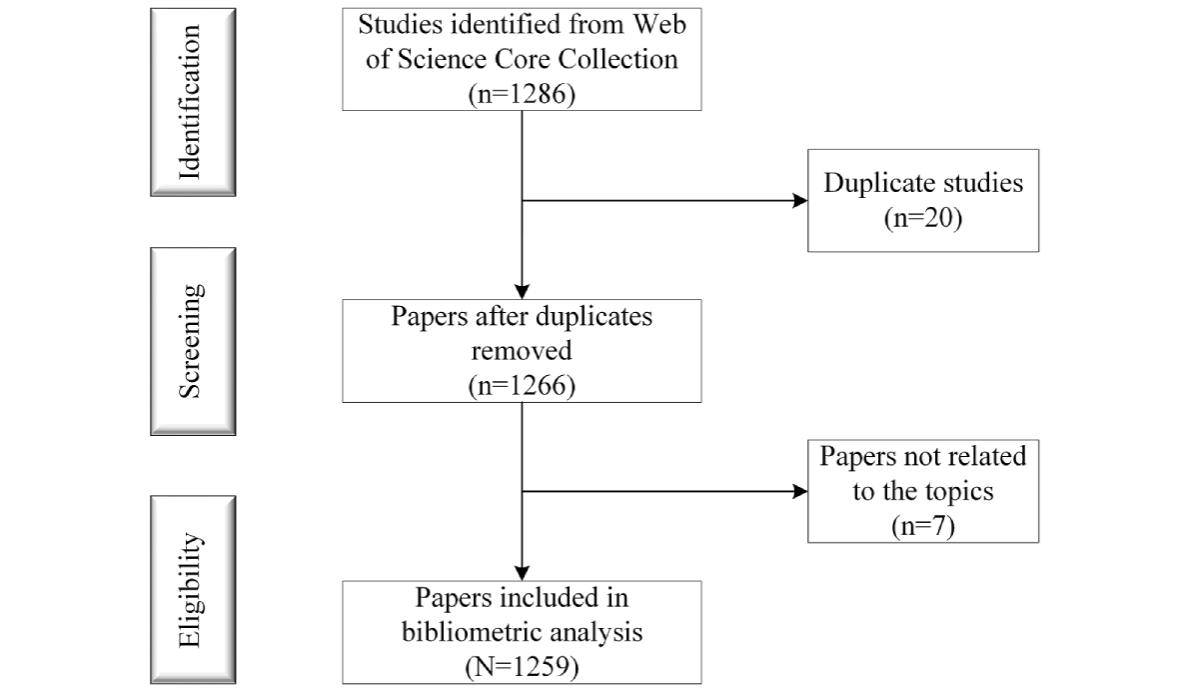
Flowchart of literature selection.

WoS generates and provides keywords plus for research publications. Keywords plus are words and phrases that are generated using algorithms based on the titles referenced or cited in the documents. WoS-assigned keywords plus express the knowledge structure of the discipline and interconnectedness of different research areas [21]. During the literature selection process, the titles, abstracts, keywords, and keywords plus of the articles were used to accept or remove published material. Articles that did not contain content related to cognition and VR in these parts were considered to be irrelevant to the topic. The author, title, publication year, and journal were used to identify duplicate records. In total, 20 duplicate records and 7 irrelevant papers were identified and removed. A total of 1259 articles were exported in the form of a full record and cited references, saved as plain text files, and stored in download_txt format (Figure 1).

### Data Analysis

In this study, CiteSpace (version 5.8.R2; Drexel University), Scimago Graphica (version 1.0.16; Scimago lab), and gCLUTO (version 1.0; Kerapis lab) software were used to perform the bibliometric visual analysis. CiteSpace was used to analyze the distribution of countries or regions, authors, journals, and cocited references. Scimago Graphica was used to generate geographic visualization maps of the countries or regions. gCLUTO was used for the keyword clustering analysis. Repeated bisection was chosen as the clustering method. The similarity was calculated by choosing the cosine function. The clustering criterion function was set to *I*^2^. The clustering results with a high ISim and low ESim were selected [22], and the number of clusters was adjusted.

### Ethical Considerations

No application for an ethical permit was submitted for this paper. According to the Chinese Hospital Association, an ethics review was not required for this secondary analysis of published data.

## Results

### The Annual Trends of Publications

**Figure 2:**
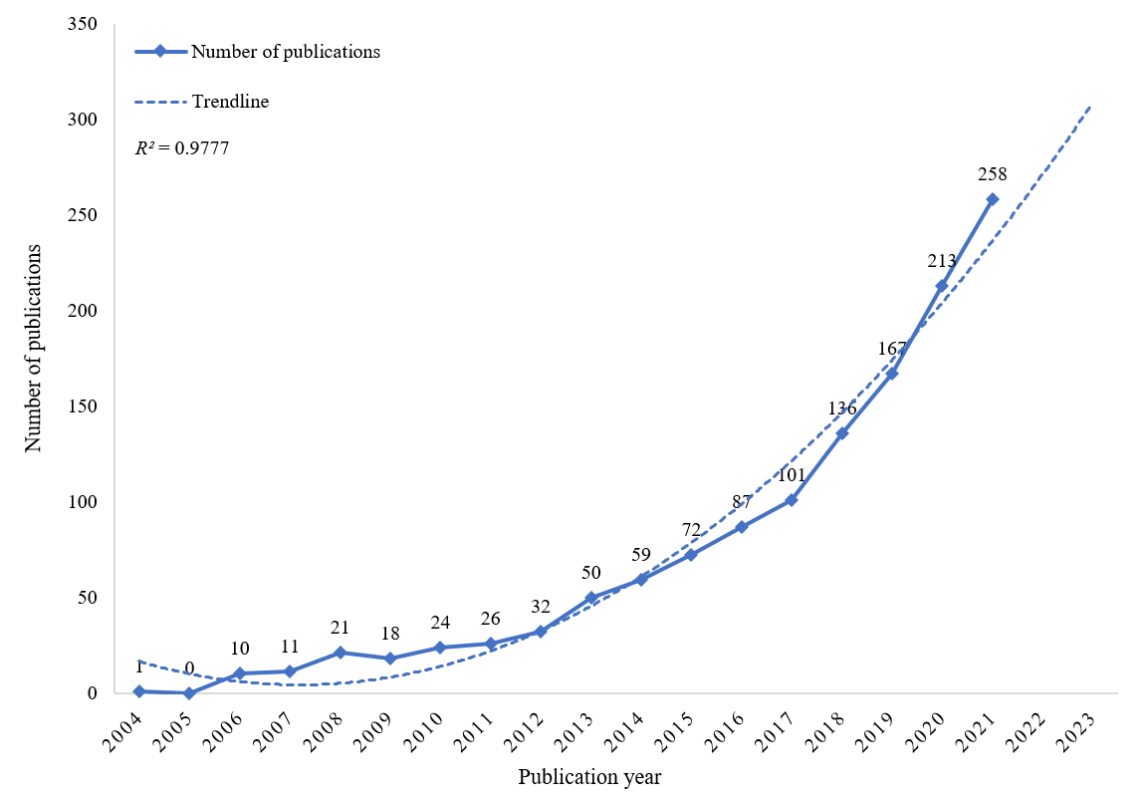
Annual publication outputs and the model fitting curve of time trend of virtual reality in cognitive rehabilitation.

The number of documents published in each period reflects the development trend of research in the field. Figure 2 plots the distribution of annual research publications on the application of VR in cognitive rehabilitation in the past 20 years.

Microsoft Excel 2019 was used to conduct linear regressions using the Trendline function and polynomials. The dotted lines in Figure 2 are polynomial fitting created by the application, which predict future publication trends in this field. The trendline equation is y = 1.22x^2^ – 10.17x + 25.54. In this equation, y represents the number of publications, and x represents the ID of publication years in temporal order. Model fitting curve revealed a positive trend in annual publication numbers over the past 20 years (*R*^2^=0.977; the closer *R*^2^ is to 1, the better the fit of the trendline). The trend line shows that the number of publications will continue to increase in the future.

According to the number of publications, the publication year can be divided into 3 periods: from 2004 to 2011, from 2012 to 2016, and from 2017 to 2021. As shown in Figure 2, no relevant papers were published before 2004. From 2004 to 2016, the number of articles in this field increased annually; however, the growth rate was relatively slow. The number of publications in 2017 reached 101, surpassing 100 for the first time. From 2017 to 2021, the number of published articles increased significantly, indicating that an increasing number of scholars have begun to focus on the potential of VR in the field of cognitive rehabilitation.

### Popular Research Themes

Each publication indexed in the WoS is associated with one or more subject categories (SCs). We identified the top 10 SCs (Table 1). The most popular research areas were neurosciences and neurology (n=392), followed by psychology (n=314), computer science (n=131), and rehabilitation (n=125). SC “computer science” accounted for only 5.2% (7/134) of publications during period 1 but increased to 10.3% (93/907) during period 3. SC “health care sciences and services” accounted for 0.8% (1/134) of publications during period 1 and then increased to about 4.9% (44/907) during period 3.

The 10 most frequently studied diseases are listed in Table 2. Aging is listed in the table because human aging is usually accompanied by typical structural and neurophysiological changes in the brain and varying degrees of cognitive decline [23]. Reviews that aimed to investigate the status of VR aid treatment for neurological or psychiatric disorders were not included in the statistics because they did not investigate a specific disease. Stroke, dementia, and mild cognitive impairment (MCI) were the 3 major research areas of the publications.

**Table 1:** The number of publications in the top 10 Web of Science (WoS) subject categories for the total study period and for each period.

WoS subject categories	Total (N=1416), n (%)	Period 1 (n=134), n (%)	Period 2 (n=375), n (%)	Period 3 (n=907), n (%)
Neurosciences and neurology	392 (27.7)	35 (26.1)	109 (29.1)	248 (27.3)
Psychology	314 (22.2)	32 (23.9)	84 (22.4)	198 (21.8)
Computer science	131 (9.2)	7 (5.2)	31 (8.3)	93 (10.3)
Rehabilitation	125 (8.9)	16 (11.9)	36 (9.6)	73 (8.1)
Psychiatry	124 (8.8)	17 (12.7)	34 (9.1)	73 (8.1)
Engineering	110 (7.8)	7 (5.2)	24 (6.4)	79 (8.7)
Geriatrics and gerontology	71 (5.0)	6 (4.5)	21 (5.6)	44 (4.9)
Health care sciences and services	54 (3.9)	1 (0.8)	9 (2.4)	44 (4.9)
Education and educational research	48 (3.4)	7 (5.3)	11 (2.9)	30 (3.3)
Behavioral science	47 (3.4)	6 (4.5)	16 (4.2)	25 (2.8)

**Table 2:** Top 10 most frequently studied diseases in virtual reality–based cognitive rehabilitation (N=1259).

Ranking	Disease	Publication, n (%)
1	Stroke	104 (8.26)
2	Dementia	91 (7.23)
3	Mild cognitive impairment	83 (6.59)
4	Aging	75 (5.96)
5	Brain injury	64 (5.08)
6	Schizophrenia spectrum and other psychotic disorders	60 (4.77)
7	Parkinson disease	45 (3.57)
8	Multiple sclerosis	22 (1.75)
9	Autism spectrum disorder	20 (1.59)
10	Posttraumatic stress disorder	11 (0.87)

### Distribution of Countries or Regions

**Figure 3:**
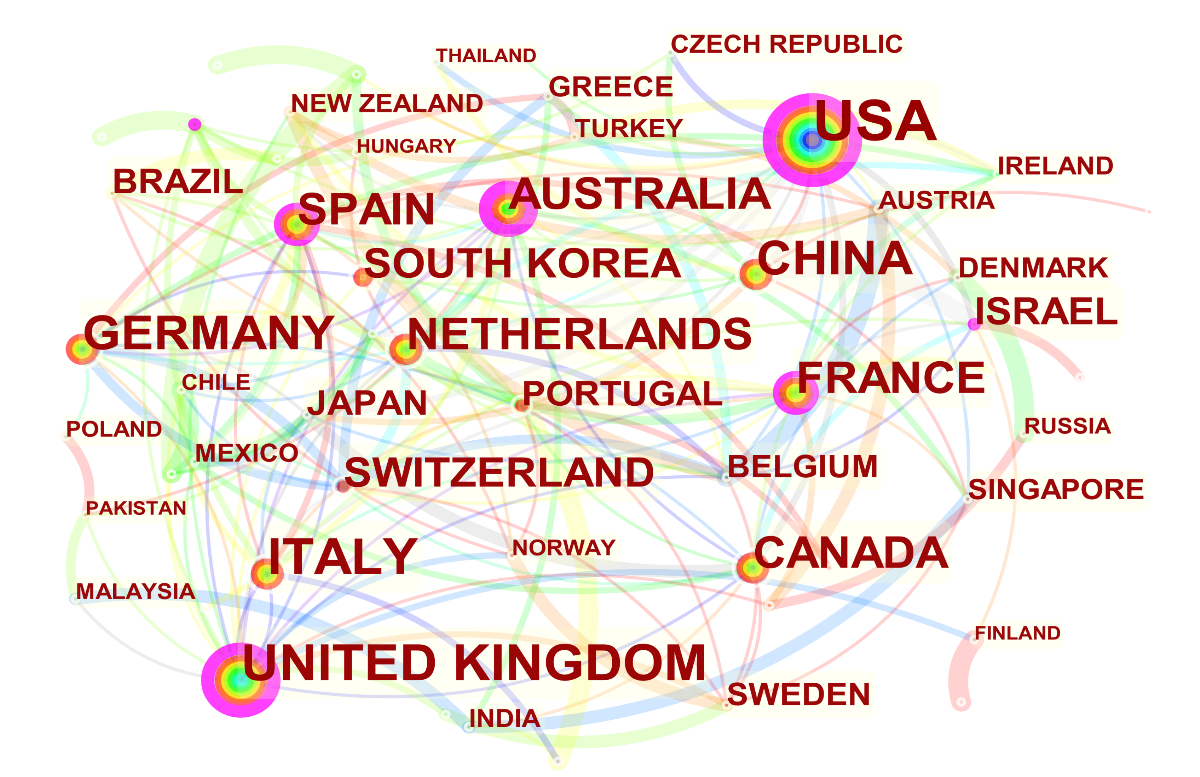
Distribution of publications from different countries.

The data were imported into CiteSpace, “Country” was selected as a node, the number of objects in each time slice was set to 50, and the minimum spanning tree algorithm was used to draw the countries or regions cooperation network map (Figure 3). A total of 1259 articles were published in 62 countries or regions. The size of the node label represents the number of papers published in the country or region. The larger the node font, the higher the number of papers published. The purple outer circle of the node indicates that the node has a high centrality. The centrality of a node is a graph-theoretical property that measures the importance of the node’s position in a network. A commonly used centrality metric is betweenness centrality. CiteSpace helped identify pivotal points by measuring the betweenness centrality of a node. It quantifies the probability that the node is on an arbitrary shortest path in the graph. Nodes with high betweenness centrality tend to be found in paths connecting different specialties or tipping points in a network [24]. The betweenness centrality of a node *v* is defined as follows as g(v):

g(v) =∑*_s≠v≠t_* σ*_st_*(v) / σ*_st_*
**(1)**

where σ*_st_* is the total number of shortest paths from node *s* to node *t* and σ*_st_*(v) is the number of shortest paths from *s* to *t* going through *v* [25].

As shown in Table 3, in the field of VR on cognitive rehabilitation, the most significant number of publications came from the United States (328/1259, 26.05%), followed by Italy (140/1259, 11.12%), the United Kingdom (130/1259, 10.33%), China (115/1259, 9.13%), and Germany (101/1259, 8.02%). These 5 countries published 64.65% (814/1259) of the total number of articles. Among the top 10 countries, the United States, the United Kingdom, Australia, Spain, France, Germany, and Canada showed a high degree of centrality.

**Figure 4:**
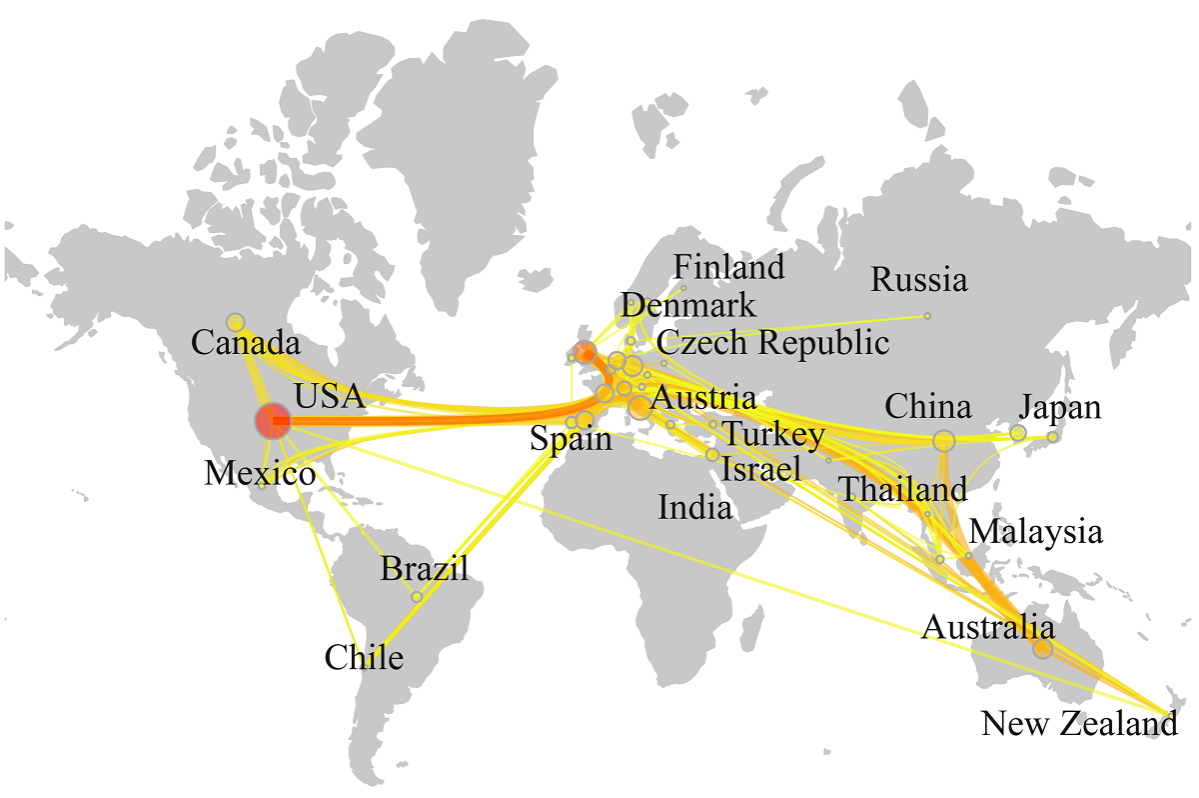
Geographic locations of countries with more than 5 publications.

Scimago Graphica software was used to analyze the countries with more than 5 publications to generate a geographic visualization map (Figure 4). The lines in the figure represent cooperation between countries, and the edge width of the lines represents the intensity of cooperation. It can be seen that the United States, the United Kingdom, Australia, China, and other countries have actively cooperated with other countries. Moreover, many European scholars have shown interest in the application of VR in cognitive rehabilitation and have conducted international cooperation to a certain extent.

**Table 3:** Countries with the top 10 publications on virtual reality in cognitive rehabilitation (N=1259).

Ranking	Country	Publication, n (%)	Centrality
1	United States	328 （26.05）	0.36
2	Italy	140 （11.12）	0.06
3	United Kingdom	130 （10.33）	0.35
4	China	115 （9.13）	0.09
5	Germany	101 （8.02）	0.1
6	Australia	90 （7.15）	0.2
7	Spain	81 （6.43）	0.18
8	Canada	81 （6.43）	0.1
9	France	80 （6.35）	0.15
10	The Netherlands	75 （5.96）	0.05

### Authors and Cooperative Relationships

**Figure 5:**
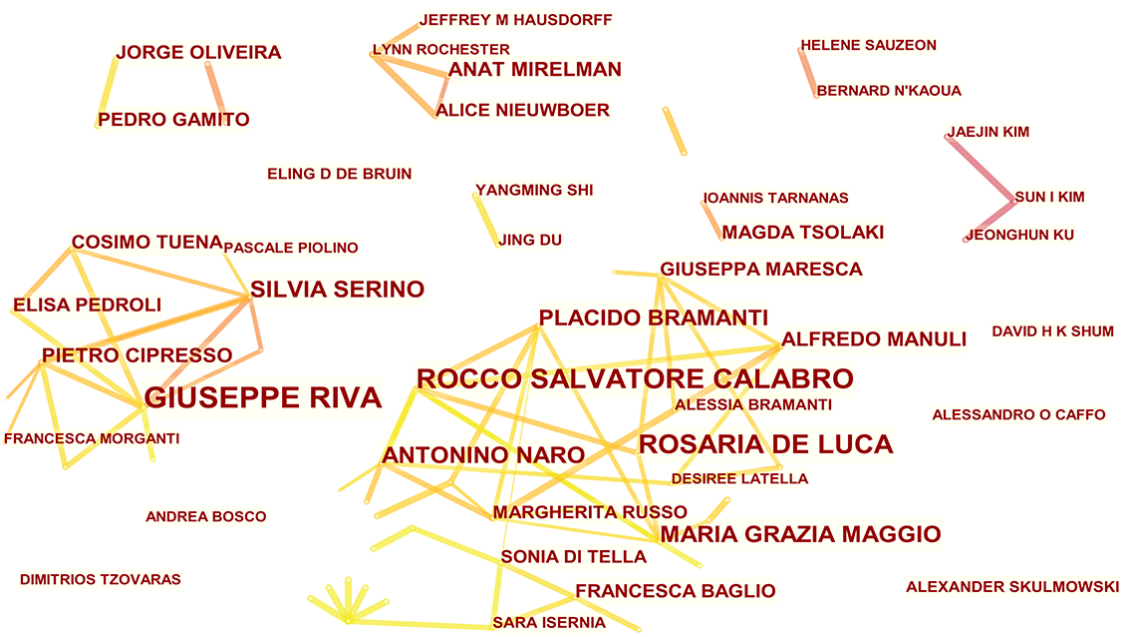
Visualization map of authors involved in the application of virtual reality in cognitive rehabilitation.

A total of 537 authors have published articles on the application of VR in cognitive rehabilitation (Figure 5). Giuseppe Riva was the most prolific author, with 2.3 (29/1259) of articles, followed by Rocco Salvatore Calabro with 1.91 (24/1259) of articles. Rocco Salvatore Calabro (24/1259, 1.91%), Rosaria De Luca (20/1259, 1.59%), Maria Grazia Maggio (14/1259, 1.11%), and Antonino Naro (14/1259, 1.11%) conducted active cooperation and communication, forming the largest author collaboration network. It is worth noting that the centrality of the top 10 authors was 0 (Table 4), suggesting that the influence of the authors on the of VR in cognitive rehabilitation needs to be improved.

**Table 4:** Top 10 productive authors on the application of virtual reality in cognitive rehabilitation (N=1259).

Ranking	Author	Publication, n (%)	Centrality
1	Giuseppe Riva [9,12,26,27]	29 （2.3）	0
2	Rocco Salvatore Calabro	24 （1.91）	0
3	Rosaria De Luca	20 （1.59）	0
4	Maria Grazia Maggio [28]	14 （1.11）	0
5	Antonino Naro [28]	14 （1.11）	0
6	Silvia Serino [26,27]	13 （1.03）	0
7	Placido Bramanti	12 （0.95）	0
8	Anat Mirelman	9 （0.71）	0
9	Alfredo Manuli [28]	8 （0.64）	0
10	Pietro Cipresso	8 （0.64）	0

### Cocited Academic Journals

**Figure 6:**
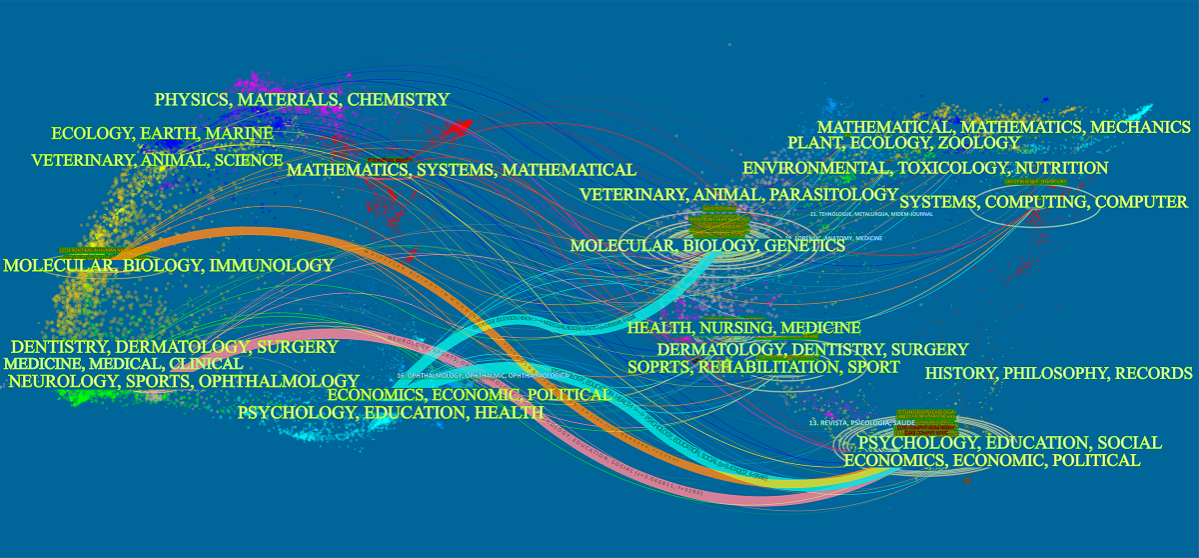
The dual-map overlay of journals on the application of virtual reality in cognitive rehabilitation.

The journal cocitation analysis revealed the overall structure of the subject and the characteristics of a journal. As shown in Table 5, the most cited journal is *Plos One* (466/28,765, 1.62%). Among the top 10 academic journals, the highest impact factor is neurology (impact factor 9.910). Furthermore, it can be seen that half of the journals in the table belong to quartile ranking position 1. The dual-map overlay of journals represents the topic distribution of the academic journals. In the dual map, the map of the citing journals is on the left, the map of the cited journals is on the right, and the colored paths between them suggest the cited relationships. There are 5 main citation paths, with 2 blue paths, 2 pink paths, and 1 orange path (Figure 6), representing that the documents published in psychology, education, or social journals are often cited by psychology, education, health, neurology, sports, ophthalmology, molecular biology, or immunology journals, and the documents published in molecular biology or genetics journals are often cited by psychology, education, health, neurology, sports, or ophthalmology journals.

**Table 5:** Top 10 cocited journals that published articles on the application of virtual reality in cognitive rehabilitation (N=28,765).

Ranking	Cocited journal	Citation, n (%)	Impact factor based on Clarivate Analytics Journal Citation Report (2020)	JCR^a^
1	Plos One	466 (1.62)	3.240	Q^b^2
2	Neuropsychologia	377 (1.31)	3.139	Q1
3	Frontiers in Human Neuroscience	353 (1.23)	3.169	Q3
4	Cyberpsychology Behavior and Social Networking	349 (1.21)	4.157	Q1
5	Frontiers in Psychology	319 (1.11)	2.988	Q2
6	Neurology	318 (1.11)	9.910	Q1
7	Archives of Physical Medicine and Rehabilitation	305 (1.06)	3.966	Q1
8	Neuroscience	295 (1.03)	3.590	Q2
9	Neuroimage	268 (0.93)	6.556	Q1
10	Presence: Teleoperators and Virtual Environments	265 (0.92)	0.597	Q4

^a^JCR: Clarivate Analytics Journal Citation Report.

^b^Q: quartile ranking position.

### Cocited References

As a statistical approach to detecting research trends, cocitation refers to the phenomenon in which 2 or more articles are cited by other articles at the same time. When a group of authors cite a common set of documents, these cocitations indicate documents that may contain concept symbols [29]. Cited articles are the foundations upon which current research is being conducted; they represent the intellectual base [30,31]. These publications may contain foundational theories, groundbreaking early works, and methodological principles in the field [31]. Among the 821 cocited references retrieved, Table 6 shows the 10 most frequently cited references, of which “Using Virtual Reality to Characterize Episodic Memory Profiles in Amnestic Mild Cognitive Impairment and Alzheimer’s Disease: Influence of Active and Passive Encoding” is the most frequently cited (n=26).

**Table 6:** Top 10 cocited references on the application of virtual reality in cognitive rehabilitation.

Ranking	Reference	Number of citation	Centrality
1	Using Virtual Reality to Characterize Episodic Memory Profiles in Amnestic Mild Cognitive Impairment and Alzheimer’s Disease: Influence of Active and Passive Encoding	26	0
2	Cognitive Training on SStroke Patients via Virtual Reality-Based Serious Games	25	0.02
3	A Feasibility Study With Image-Based Rendered Virtual Reality in Patients With Mild Cognitive Impairment and Dementia	23	0.04
4	Computerized and Virtual Reality Cognitive Training for Individuals at High Risk of Cognitive Decline: Systematic Review of the Literature	23	0.06
5	The Effectiveness of Virtual Reality for People With Mild Cognitive Impairment or Dementia: a Meta-analysis	22	0.01
6	Benefits of Virtual Reality-Based Cognitive Rehabilitation Through Simulated Activities of Daily Living: a Randomized Controlled Trial With Stroke Patients	22	0.07
7	A Succinct Overview of Virtual Reality Technology Use in Alzheimer’s Disease	22	0.14
8	Effects of Virtual Reality-Based Physical and Cognitive Training on Executive Function and Dual-Task Gait Performance in Older Adults With Mild Cognitive Impairment: a Randomized Control Trial	21	0
9	Effects of Virtual Reality-Based Training With BTs-Nirvana on Functional Recovery in Stroke Patients: Preliminary Considerations	20	0.01
10	Virtual Reality in the Assessment, Understanding, and Treatment of Mental Health Disorders	20	0.03

### Distribution of Keywords

By analyzing the keywords, we can summarize the study topics in a specific field and explore hot spots. Data cleaning was performed to remove coding errors, because different articles may use different keywords to refer to the same concept. For example, “older people,” “elderly,” and “old adult” represent the same concept, they were recorded as “old adult,” and “cognitive impairment” and “cognitive dysfunction” were combined into the keyword “cognitive impairment.” In all 1259 publications, we obtained 544 keywords. The keyword “virtual reality” has the highest frequency of 851. Other keywords with high frequency include “cognition” (n=236), “rehabilitation” (n=193), “performance” (n=164), “older adult” (n=151), “memory” (n=143), “dementia” (n=143), “Alzheimer’s disease” (n=141), “mild cognitive impairment” (n=125), and “environment” (n=123; Table 7).

CiteSpace burst-detection algorithms were adapted for detecting sharp increases in interest in a specialty [24]. According to keyword burst detection, Table 8 lists the top 20 keywords with the strongest citation bursts. The red line represents the duration of the burstness. Among these words, “social cognition” (6.22) was the strongest burst keyword during the period between 2004 and 2021, followed by “environment” (6.00), “brain injury” (5.86), “spatial memory” (5.55), and “episode memory” (5.51).

**Table 7:** Top 10 keywords related to the application of virtual reality in cognitive rehabilitation.

Ranking	Keyword	Frequency	Centrality
1	Virtual reality	851	0.06
2	Cognition	236	0.1
3	Rehabilitation	193	0.04
4	Performance	164	0.17
5	Older adult	151	0.05
6	Memory	143	0.07
7	Dementia	143	0.04
8	Alzheimer’s disease	141	0.06
9	Mild cognitive impairment	125	0.04
10	Environment	123	0.09

**Table 8:** Top 20 keywords with the strongest citation burst related to the application of virtual reality in cognitive rehabilitation.

Ranking	Keywords	Strength	Start year	End year	2004-2021
1	Brain injury	5.86	2006	2013	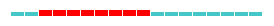
2	Environment	6.00	2006	2009	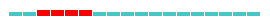
3	fMRI^a^	4.14	2007	2015	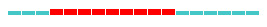
4	Strategy	3.45	2007	2015	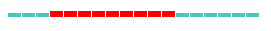
5	Performance	3.41	2007	2012	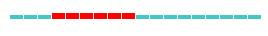
6	Spatial memory	5.55	2008	2014	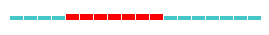
7	Skill	3.45	2009	2014	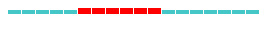
8	Cognitive function	4.78	2009	2013	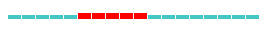
9	Hippocampus	5.35	2010	2017	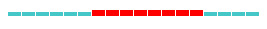
10	Ecological validity	3.52	2013	2017	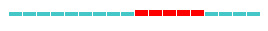
11	Neuropsychological assessment	3.88	2013	2016	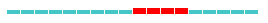
12	Human	3.85	2013	2015	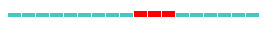
13	Spectrum disorder	3.65	2013	2014	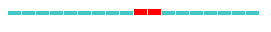
14	Social cognition	6.22	2014	2017	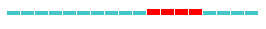
15	Prefrontal cortex	3.99	2014	2017	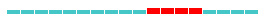
16	Episodic memory	5.51	2015	2017	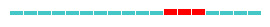
17	Serious game	3.59	2017	2018	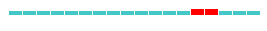
18	Randomized controlled trial	3.40	2018	2019	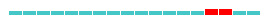
19	Telerehabilitation	3.36	2019	2021	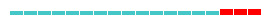
20	Recognition	3.34	2019	2021	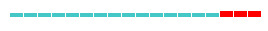

^a^fMRI: functional magnetic resonance imaging.

**Figure 7:**
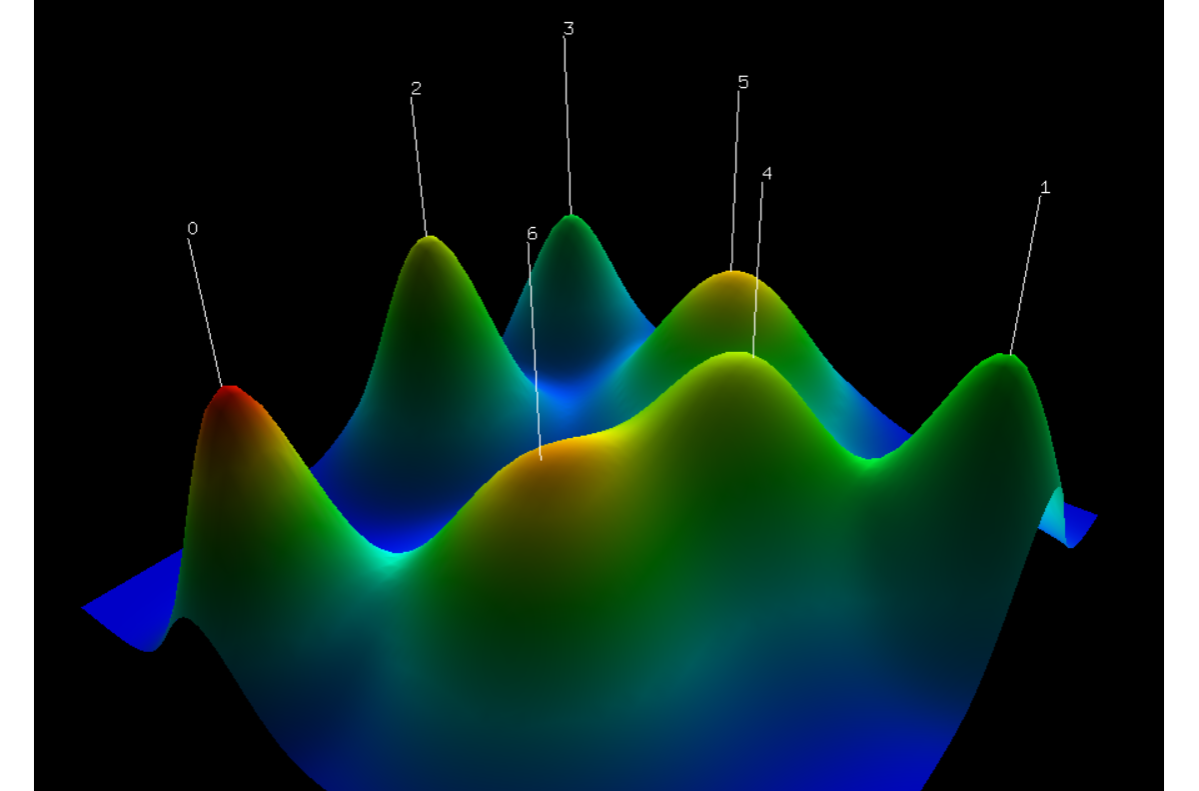
Keyword clustering hill plot of publications related to the application of virtual reality (VR) in cognitive rehabilitation.

**Figure 8:**
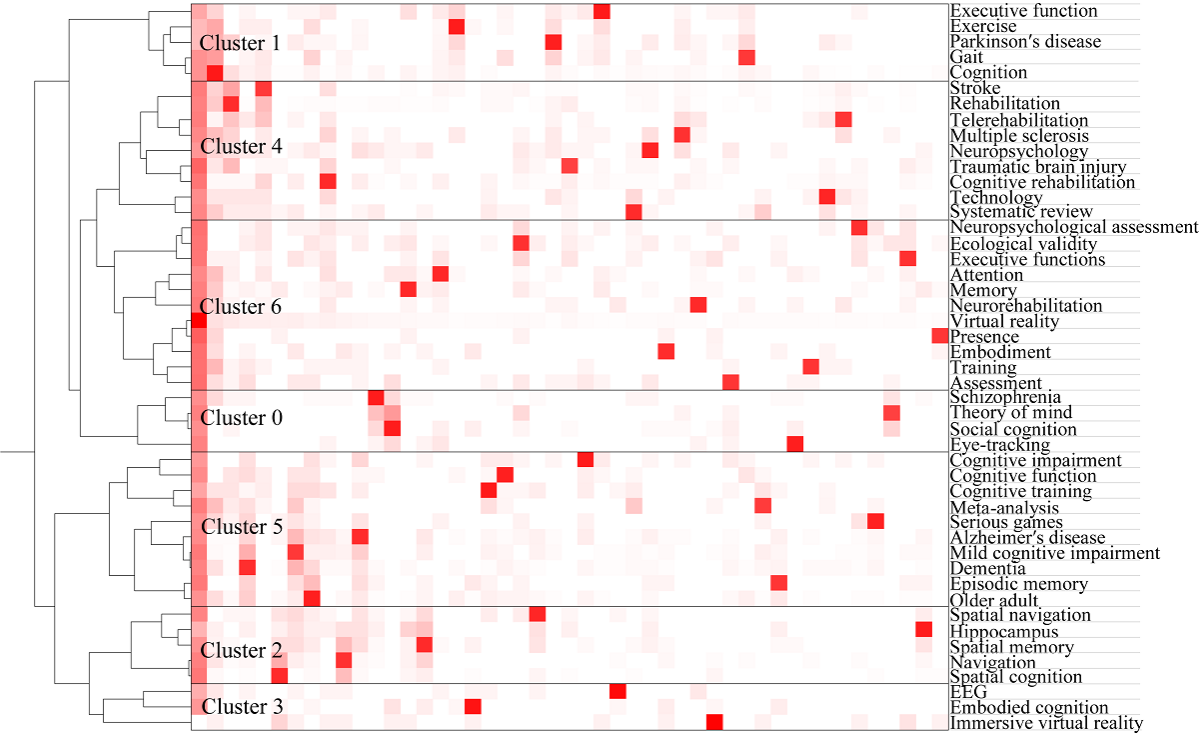
Clustering dendrogram of keywords of publications related to the application of virtual reality in cognitive rehabilitation. EEG: electroencephalogram.

Different clusters can be formed based on the keywords with different degrees of closeness. Identifying these clusters can provide an intuitive understanding of various hot subfields of research in a particular field. The keyword co-occurrence matrix was imported into gCLUTO for clustering analysis. A keyword clustering hill plot was generated (Figure 7). Each hill represents a cluster, and there are 7 clusters. The shape of the hill is a Gaussian curve. The height of the hill is proportional to the similarity of the documents within the cluster, while the volume of the hill is proportional to the number of documents contained within the cluster. Only the color of the top of the hill is meaningful, with a red bias representing a low intraclass SD and a blue bias representing a high intraclass SD [22]. The dendrogram from clustering shows that the hot spots of research on the application of VR in cognitive rehabilitation are focused on MCI in older adults, Alzheimer disease (AD), stroke, and traumatic brain injury (Figure 8). Cluster 6 was the largest cluster and contained 11 keywords. The high-frequency keywords were “neuropsychological assessment,” “presence,” “embodiment,” and “virtual reality.”

**Figure 9:**
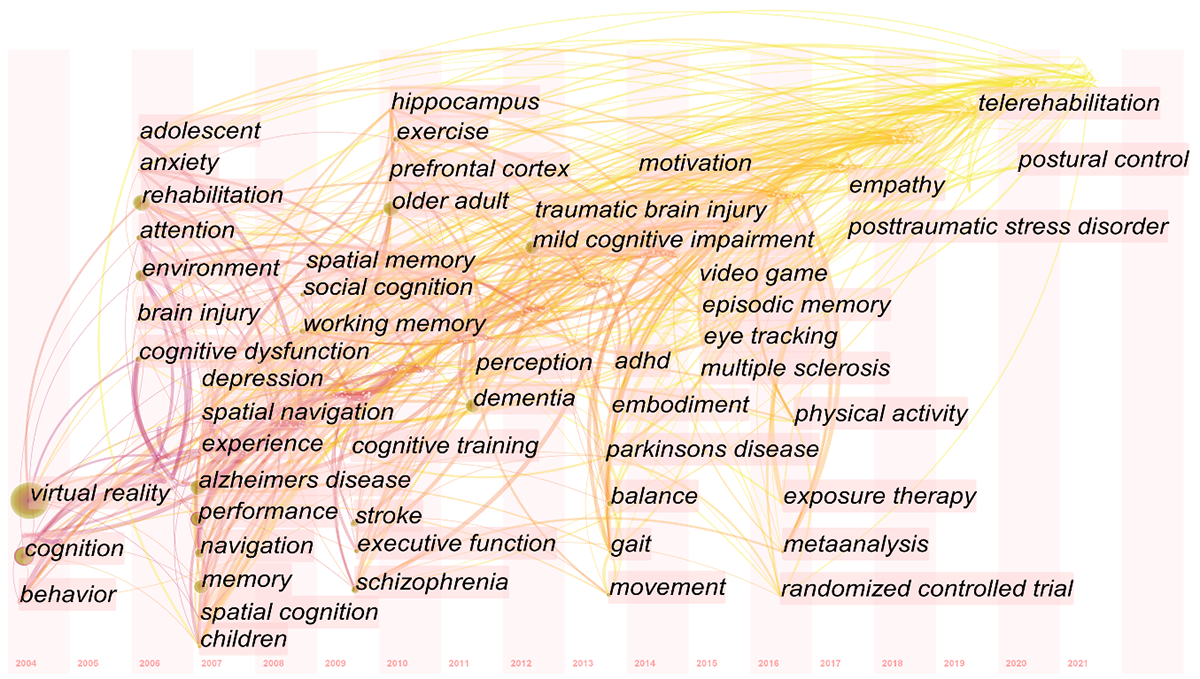
The keywords time-zone view of publications related to the application of virtual reality in cognitive rehabilitation.

A time-zone view can help show changes in the research trends in a field over time. From the time-zone view, nodes in the same period can be gathered in the same time zone. The year represents the time when the keyword first appears. The link between keywords means that they appear in the same document. It can be seen from Figure 9 that there is no relevant document was published before 2004. Keywords appeared more intensively from 2006 to 2016, but most were related to diseases, such as “brain injury,” “depression,” “Alzheimer’s disease,” and “attention deficit hyperactivity disorder (ADHD).” Keywords related to psychology and psychiatry, such as “anxiety,” “schizophrenia,” and “depression,” appeared in the early stages of VR-based cognitive rehabilitation development. As time goes on, research hot spots such as “telerehabilitation” and “post-traumatic stress disorder” emerged.

### Institutions

A total of 65.7% (830/1259) of documents on VR-based cognitive rehabilitation were funded. The top 10 funding institutions by the number of publications are listed in Table 9. Half of the top 10 funding support institutions are from the United States, indicating that the United States has strong support and substantial funding for research in this field.

Table 10 presents the research areas funded by these institutions. We noted that they tend to fund multiple disease areas rather than a single disease. The National Institute on Aging (NIA) seems to place greater emphasis on research in neurosciences and neurology and geriatrics and gerontology. Similarly, research in psychiatry is more likely to be funded by National Institute of Mental Health (NIMH).

**Table 9:** Funding institutions for publications related to the application of virtual reality in cognitive rehabilitation (N=830).

Rank	Funding	Country	Publication, n (%)
1	United States Department of Health Human Services	United States	82 (9.9)
2	National Institutes of Health United States	United States	82 (9.9)
3	European Commission	European Union	75 (9)
4	National Science Foundation	United States	43 (5.2)
5	National Natural Science Foundation of China	China	30 (3.6)
6	German Research Foundation	German	25 (3)
7	National Institute of Mental Health	United States	23 (2.8)
8	United Kingdom research innovation	United Kingdom	19 (2.3)
9	Canadian Institutes of Health Research	Canada	19 (2.3)
10	National Institute on Aging	United States	18 (2.2)

**Table 10:** Top 10 funding institutions and research area on virtual reality in cognitive rehabilitation.

	HHS^a^ (n=92), n	NIH USA^b^ (n=91), n	European Commission (n=94), n	NSF^c^ (n=43), n	NSFC^d^ (n=29), n	DFG^e^ (n=27), n	NIMH^f^ (n=27), n	UKRI^g^ (n=23), n	CIHR^h^ (n=24), n	NIA^i^ (n=22), n	Total (n=472), n
Neurosciences and neurology	31	32	31	4	9	5	8	9	8	9	146
Psychology	21	21	18	13	3	10	5	7	7	2	107
Computer science	1	1	7	11	7	4	1	0	0	0	32
Rehabilitation	9	10	6	2	1	1	0	0	2	4	35
Psychiatry	12	12	10	0	1	1	10	3	1	1	51
Engineering	3	0	11	9	5	3	0	1	1	0	33
Geriatrics and gerontology	4	4	2	1	1	0	0	1	2	4	19
Health care sciences and services	4	4	0	0	0	2	1	0	1	1	13
Education and educational research	2	2	1	2	0	0	1	1	0	0	9
Behavioral sciences	5	5	8	1	2	1	1	1	2	1	27

^a^HHS: United States Department of Health and Human Services.

^b^NIH USA: National Institutes of Health.

^c^NSF: National Science Foundation.

^d^NSFC: National Natural Science Foundation of China.

^e^DFG: German Research Foundation.

^f^NIMH: National Institute of Mental Health.

^g^UKRI: UK Research Innovation.

^h^CIHR: Canadian Institutes of Health Research.

^i^NIA: National Institute on Aging.

## Discussion

### General Information

According to the annual publication output, the publication years could be divided into 3 phases. The period from 2004 to 2011 could be considered the first phase. The number of publications increased slowly during this stage. The period 2012 to 2017 could be seen as the second phase. In 2012, Google unveiled “Project Glass,” with the promise to deliver an augmented reality head-mounted display device to the masses. Oculus released the first working prototypes of the Oculus Rift VR headset. Both the quality and availability of VR hardware and software components have changed significantly during this time. Thus, period 2 and period 3 demonstrate an increased interest in computer science. In the last phase (2017-2021), there was a sharp growth in publications. Trends in home computing and entertainment made user-friendly, robust, and polished extended VR systems available for personal ownership in the 2010s, accelerating the pace of growth in VR telehealth research [32]. Since then, remote health care apps have developed significantly, such as VR tele–mental health, telemonitoring of fully remote interventions, and specialized medical equipment with VR and augmented reality [33]. These results suggest that research is becoming more mature, and researchers have developed considerable interest in the application of VR in cognitive rehabilitation.

VR-based cognitive rehabilitation contributions come from all over the world, including the United States, Italy, the United Kingdom, China, Germany, Australia, Spain, Canada, France, and the Netherlands taking positions of prominence. China is the only Asian country among them, which indicates that scholars from Asian countries have not attached much importance to research in this field. In contrast, funders and scholars in the United States have shown continued research interest. A bibliometric analysis of VR and health care have similarly demonstrated that the United States was the country with the highest contribution (26.4% vs 26.1% in this report) [34]. Among the top 10 contributing countries, the United States is the most prominent in terms of funding for scientific research. According to the last census of the population, nearly 1 in 5 people have a disability in the United States. By 2050, the number of individuals with disabilities is estimated to exceed 64 million. With more people who experience disabling health events (eg, stroke or traumatic brain injury) surviving and living longer, the number may be even higher [35].

Over the past 20 years, several reimbursement methods for medical rehabilitation have been developed in the United States. One important transition was the inclusion of “rehabilitative and habilitative services and devices” as one of the 10 “essential benefits” of the Affordable Care Act in 2010 [35]. In April 2013, a number of public and private institutions and agencies joined together to launch Brain Research Through Advancing Innovative Neurotechnologies (BRAIN), with the NIH (part of the United States Department of Health & Human Services) playing an important role. The BRAIN aims to help researchers to seek new ways to treat, cure, and even prevent brain disorders. By 2021, the NIH has awarded more than 1100 awards to hundreds of researchers, totaling approximately US $2.4 billion [36]. Owing to the cross-cutting nature of this project, the NIH BRAIN Initiative is managed by 10 NIH institutes and centers, including the NIA and NIMH listed in this paper.

In the field of pharmaceutical science, 67% to 97% of drug development is conducted by the private sector [37]. However, we note that most funded studies identified in this paper are supported by public institutions (NIH, National Science Foundation, Canadian Institutes of Health Research, German Research Foundation, National Natural Science Foundation of China, UK Research Innovation, and European Commission; the NIA and NIMH are both institutes and centers that make up the NIH, which is a part of the United States Department of Health and Human Services). This finding may be because some private companies prefer to provide the supply of VR products or devices rather than financial support. A second explanation may be that private industries that develop and manufacture VR products do not have the means to fund research in the VR-based cognitive rehabilitation field fully on a sufficiently large scale. A third way to understand these results is that market risk and uncertainty are too high to elicit private investment. According to Sharma et al [38], VR technology is still in its early stages, and obtaining funds from investors is one of the most significant problems because predicting the level of public acceptance is difficult. It is clear that more research is needed to analyze the reasons for this variation between types of funders and their impact.

Among prolific authors, Giuseppe Riva, an Italian academic, has the highest number of publications. Over the past decade, his research on VR-based cognitive rehabilitation has focused on various neurodegenerative disorders such as AD, Parkinson disease (PD), and MCI. A previous bibliometric analysis of VR research in MCI by Zhu et al [39] similarly demonstrated that Giuseppe Riva is the most popular scholar. The other 3 authors in the top 10 list of this study were also in the top 10 list of Zhu et al [39]; they are Silvia Serino, Anat Mirelman, and Pietro Cipresso. It is worth noting that the top 10 authors have a centrality of 0, which indicates that their influence in this research field needs to be further improved. Rocco Salvatore Calabro, Rosaria De Luca, Maria Grazia Maggio, Antonino Naro, Placido Bramanti, and Alfredo Manuli have published articles in the VR-based cognitive rehabilitation field as a research team. Therefore, the research topics of these authors are highly similar. Similarly, Giuseppe Riva, Silvia Serino, and Pietro Cipresso are from Milan, Italy; they formed a cohesive team. Moreover, Anat Mirelman , a professor at Sackler School of Medicine and Sagol School of Neuroscience at Tel Aviv University, ranked eighth. Her research interests in VR-based cognitive rehabilitation are in understanding aging and neurodegenerative diseases such as PD. The research topics of the authors in the top 10 list of this study are relatively concentrated, which implies that cooperation between different research teams was not close. Cross-cooperation between researchers with different disciplinary backgrounds can promote the progress of a certain research subject [40]. We suggest that awareness of this discrepancy may encourage researchers to share knowledge and experience from different disciplines.

The cocitation frequency reflects the quality and influence of the journal. Among the 10 journals with high cocitation frequency, half of the journals belong to quartile ranking position 1. These journals have an important impact on the international community, indicating that the application of VR in cognitive rehabilitation has received worldwide attention.

Through the analysis of cocited documents, it can be found that the paper with the highest citation frequency in the past 20 years is “Using Virtual Reality to Characterize Episodic Memory Profiles in Amnestic Mild Cognitive Impairment and Alzheimer’s Disease: Influence of Active and Passive Encoding.” We have mentioned that compared with the traditional language test, the VR test can test memory in a way that is closer to the memory needs of daily life [41].

### Research Hot Spots and Frontiers

Both stroke and dementia can pose a significant threat to older adults; they often frequently coexist and share certain risk and protective factors [4]. The VR tasks to assess and train episodic memory of older adults are a research hot spot in geriatrics. VR provides an immersive experience and provides more natural interactions with the surrounding environment. There is a trend in research that more immersive VR systems promote improved episodic memory performance [42]. Within this context, interest in examining presence in experiments that investigate the effects of immersion on memory in virtual environments has started to grow.

MCI is common in older populations, and its prevalence increases with age [43]. It is a significant risk factor for AD. AD is irreversible and presents with significant treatment difficulties. Early identification and intervention are critical for improving the prognosis of MCI [44,45]. Numerous randomized controlled trials have been conducted on drug therapies for MCI. However, when compared with drug therapy, VR-based interventions seem to be more cost-effective and accessible [45].

Cognitive impairment no dementia has been commonly considered to be a normal consequence of brain aging [46]. However, some subtypes of cognitive impairment no dementia often represent early dementia and affect dementia and incident disability [4]. Perhaps due to the fact that older adults with cognitive impairment no dementia may be a potential target population for VR-based cognitive training to prevent or delay the onset of dementia, this field is a research hot spot. Meanwhile, age-associated memory impairment has been of interest to scholars because of the difficulties it may engender in the performance of everyday activities.

Introducing an embodied cognition approach helps to integrate the recovery of motor functions into cognitive rehabilitation [47]. Embodied cognition states that there is a close relationship between the body and mind (or cognition). Motor programming and execution depend on the interaction between the body, cognition, and the external environment. Therefore, peculiar motor tasks contextualized in a specific environment can enhance cognitive functions [28]. Bocanegra et al [48] interpreted PD within an embodied cognitive framework. According to them, language and cognition are grounded in lower-level sensorimotor mechanisms. As Tuena et al [26] claimed, VR can influence cognitive processes as an embodied tool.

Posttraumatic brain injury (TBI) is one of the main research hot spots in the field of VR-based cognitive rehabilitation. According to a study conducted by Maas et al [49], epidemiological patterns of TBI in high-income countries are changing. TBI because of traffic-related incidents has decreased, and older age of patients with TBI because of falls is increasing. Moreover, aging is closely linked to AD, PD, MCI, and cognitive impairment with no dementia. In general, the research participants of cognitive rehabilitation based on VR are mainly older adults, regardless of whether their cognitive decline is caused by disease.

The use of immersive VR was beneficial in managing a spectrum of mental disorders, such as the treatment of persecutory delusions in the context of schizophrenia spectrum disorders [50], social adaptation skills training for children with autism spectrum disorder [51], and exposure therapy for combat-related posttraumatic stress disorder in the active-duty military [52].

VR-based rehabilitation is not a well-defined multidisciplinary field, but a network of traditional disciplines that utilize common technology [18]. Therefore, there are significant differences between VR-based rehabilitation methods. For example, VR-based exposure therapy has been used for eating disorders [53] and combat-related posttraumatic stress disorders [52]. For older people, VR-based therapies integrated with movement therapy have been found to improve spatial navigation [54]. Furthermore, some studies have applied game elements to VR-based rehabilitation such as serious games and virtual spatial wayfinding games [55,56].

Since the existing studies are diverse, more implementation studies are needed in different populations to evaluate the effectiveness and maximize the potential of VR in cognitive rehabilitation. In addition, as Birckhead et al [57] claimed, it is essential to develop and evaluate VR therapy within a common scientific framework.

### Application and Development Trend of Research

Over the past 20 years, VR has been widely used in cognitive rehabilitation for MCI, AD, PD, stroke, brain injury, and posttraumatic stress disorder. With the development of high-performance mobile computing and various software programs, highly immersive VR has become available and affordable [58]. Currently, commonly used VR systems include Kinect from the United States, Nintendo Wii from Japan, HTC Vive from Taiwan, China, and Samsung Gear VR from South Korea. Using these VR systems in cognitive rehabilitation, we expect further improvement in patient compliance and interest in therapy.

With the continuous interpersonal transmission of COVID-19, patients’ access to health care is limited, but VR technology can overcome this limitation to some extent. The VR-based telerehabilitation provides an immersive environment for the patient while allowing remote monitoring of the rehabilitation progress. Studies have shown that VR-based telerehabilitation cost less than clinic-based rehabilitation programs [59,60]. However, the safety of VR-based telerehabilitation requires further assessment because the home environment is not under the control of medical staff. VR-related side effects may cause physical risks to people who are sensitive to motion sickness or those with impaired function [61]. Therefore, future research should be devoted to assessing the safety of VR-based telerehabilitation in a large patient sample to guide clinical practice.

In this study, we found that the psychology and psychiatry field is a growing area of research in VR-based cognitive rehabilitation, which is consistent with a previous study [62]. Traditional therapeutic tools are limited to psychotherapy and drug therapy. The development of VR technology has promoted its application in this field, including a series of studies aimed at assessing and improving symptoms of schizophrenia [63], as well as VR emotion recognition tasks aimed at treating facial emotion recognition disorders in patients with neurological and psychiatric disorders in recent years [64,65]. To develop VR into a mature tool for improving mental health, researchers need to integrate knowledge from health care, computer science, neuroscience, psychology, social cognition, multisensory perception, and multimedia development in an interdisciplinary approach [27] and specifically combine user needs. This means that research on VR-based cognitive rehabilitation should consider more stakeholders such as patients, clinicians, therapists, nurses, and software developers. Better quality research is needed to explore the potential of VR technology in different disciplines and develop more patient-appropriate intervention strategies.

### Strengths and Limitations

To our knowledge, this is the first bibliometric analysis of research on VR-based cognitive rehabilitation. By including SCs, keywords, countries or regions, authors, cited journals, cited references, and funding institutions in the analysis, we were able to demonstrate a more comprehensive current situation of VR in cognitive rehabilitation. However, this study searched only 1 database, implying that it probably ignored high-quality literature in the field. In addition, the data were last updated in December 2021. As the literature in the database is constantly updated, the retrieval results of this study may differ from the actual number. The third limitation relates to the CiteSpace visual atlas. There is no standard method for setting thresholds and pruning mode in the process of generating the visual network, and as such, these elements are somewhat subjective.

### Conclusions

On the basis of the WoS database and Scimago Graphica, gCLUTO, and CiteSpace software, this study presented the research overview of the application of VR in cognitive rehabilitation scientifically and intuitively. The bibliometric analysis of articles shows that research on VR-based cognitive rehabilitation has developed intensively over the past 20 years, which is embodied in the increase in the number of papers in core journals and the enhancement of the cooperative research network between countries or regions and authors. The United States and its public foundations played a leading role in this field. This paper highlights neuroscience and neurology, psychology, computer science, and rehabilitation as the main research areas in this field. VR-based cognitive rehabilitation is mainly used to treat mental disorders, brain diseases, and nervous system diseases, but a standard treatment method has not yet been developed. Moreover, this paper raises questions that require further research regarding safety hazards in VR-based remote cognitive rehabilitation and reasons for variation between types of funders.

## Abbreviations

AD: Alzheimer disease
BRAIN: Brain Research Through Advancing Innovative Neurotechnologies
MCI: mild cognitive impairment
NIA: National Institute on Aging
NIH: National Institute of Health
NIMH: National Institute of Mental Health
PD: Parkinson disease
SC: subject category
TBI: posttraumatic brain injury
VR: virtual reality
WoS: Web of Science
